# Research progress of ectopic thyroid cancer in thyroglossal duct cyst: A case report and literature review

**DOI:** 10.1097/MD.0000000000038540

**Published:** 2024-06-28

**Authors:** Fan Bu, Kai Yu, Bingfei Dong, Wenjun Wang, Li Rong, Jixue Wang, Shuai Xue, Fang Wan, Dandan Yu, Ji Lu, Guang Chen

**Affiliations:** a Department of Thyroid Surgery, The First Hospital of Jilin University, Changchun, China; b Plastic and Aesthetic Surgery Department, The First Hospital of Jilin University, Changchun, Jilin, China; c Urology Department, The First Hospital of Jilin University, Changchun, Jilin, China; d Department of Pathology, The First Hospital of Jilin University, Changchun, Jilin, China.

**Keywords:** case report, papillary thyroid carcinoma, thyroglossal duct carcinoma, thyrohyoid carcinoma, thyrolingual cyst

## Abstract

**Rationale:**

Thyroglossal duct carcinoma, a rare clinical condition characterized by ectopic thyroid adenocarcinoma within thyroglossal duct cysts (TGDCs), typically confirmed through intraoperative rapid pathology, this condition generally has a favorable prognosis. Nevertheless, comprehensive treatment guidelines across all disease stages are lacking, the purpose of this study is to report 1 case of the disease and propose the treatment plan for each stage of the disease.

**Patient concerns:**

A patient presented with thyroid swelling, classified as C-TIRADS 4A following a physical examination. Preoperative thyroid puncture identified papillary thyroid carcinoma, and genetic testing revealed a BRAF gene exon 15-point mutation. Ancillary tests showed a slightly decreased thyroid stimulating hormone (TSH) level (0.172) with no other significant abnormalities.

**Diagnoses:**

Preoperative fine-needle aspiration cytology (FNAC) confirmed right-side thyroid cancer. Intraoperative exploration uncovered a TGDC and intraoperative rapid pathology confirmed thyroglossal duct carcinoma.

**Interventions:**

A Sistrunk operation and ipsilateral thyroidectomy were performed.

**Outcomes:**

Postoperative recovery was satisfactory.

**Lessons:**

Thyroglossal duct carcinoma is a rare disease affecting the neck. Due to limited clinical cases and the favorable prognosis associated with this condition, there is currently no established set of diagnostic and treatment guidelines. According to tumor size, lymph node metastasis, thyroid status and other factors, the corresponding treatment methods were established for each stage of thyroglossal duct cancer, which laid the foundation for the subsequent treatment development of this disease.

## 1. Introduction

During the early stages of embryonic development, the thyroid originates from the thyroid primordium. The thyroid primordium gradually descends to the anterior aspect of the thyroid cartilage, giving rise to the thyroid gland. The tubular structure formed during the descent of the thyroid primordium, connected to it, is known as the thyroglossal duct. This duct typically undergoes progressive degeneration and disappearance between the 6th and 8th weeks of gestation. In cases where incomplete degeneration of the thyroglossal duct occurs, it may lead to the formation of a thyroglossal duct cyst (TGDC). In instances where the thyroglossal duct incompletely regresses, residual thyroid tissue may persist within it. The transformation of such residual thyroid tissue can result in the development of thyroglossal duct carcinoma.^[1]^

This disease was first described in 1911.^[2]^The incidence of this condition is slightly higher in women than in men, with a ratio of 2.1:1, and it typically manifests in middle age.^[3]^ The Sistrunk procedure is the most commonly treatment in clinical practice, while the decision to perform ipsilateral thyroid and cervical lymph node dissection is based on the patient local and surrounding lymph node involvement. Overall, the prognosis of thyroglossal duct carcinoma is favorable with a low recurrence rate; however, no standardized treatment protocol has been established.

In this report, we present a rare case of thyroglossal duct carcinoma in a 56-year-old female patient. Furthermore, we review the available information on papillary thyroid carcinoma within the thyroglossal bone, summarizing its epidemiology, etiology, clinical presentation, imaging features, diagnosis, and treatment options. This article presents a novel case of thyroglossal duct carcinoma and, based on a comprehensive review of this case and previously published treatments for related diseases, proposes a treatment flowchart for the various stages of thyroglossal duct carcinoma. The purpose of this flowchart is to offer a systematic therapeutic approach for thyroglossal duct carcinoma within this field.

## 2. Case description

### 2.1. History and examination

A 56-year-old female patient sought care at the Thyroid Surgery Department of the First Hospital of Jilin University primarily due to a thyroid goiter identified during a physical examination. The patient had a well-controlled history of diabetes mellitus for 2 years. She reported no symptoms of shortness of breath, hoarseness, choking, heat intolerance, or excessive sweating. Furthermore, she had not experienced any significant weight loss in the past 3 months and exhibited good mental status. Additionally, the patient had previously undergone a myomectomy at our hospital 3 years ago and had a successful recovery.

During the preoperative auxiliary examinations of the patient, only a slight decrease of 0.172 in thyrotropin (thyroid stimulating hormone [TSH]) was observed, with no other notable abnormalities detected. Genetic testing revealed a mutation at the 15th exon point of the BRAF gene. Preoperatively, thyroid aspiration confirmed papillary carcinoma on the right side of the thyroid.

### 2.2. Imaging findings

**Figure 1.**
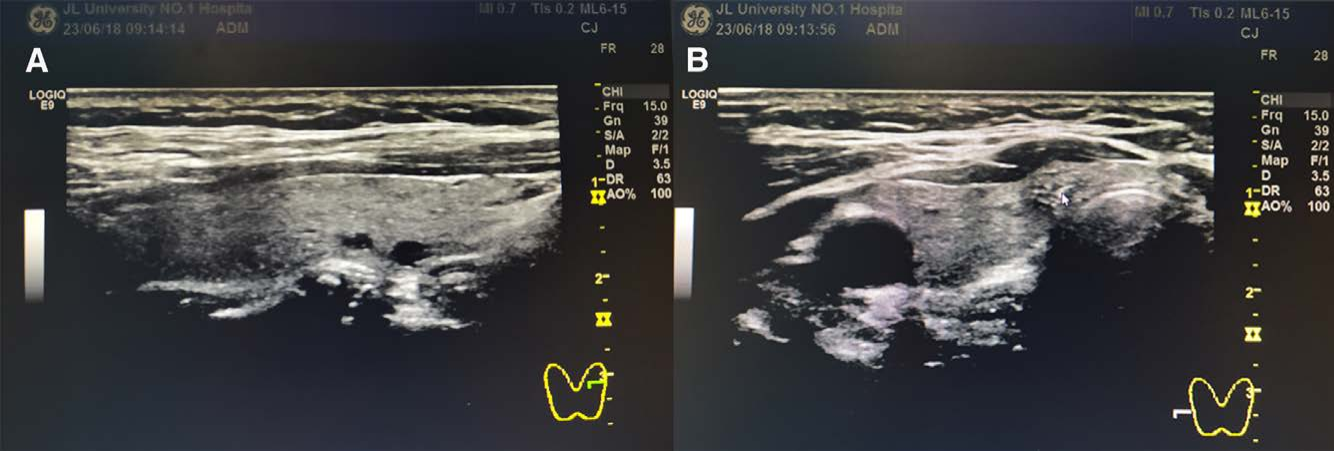
(A) Ultrasound of the left lobe of the thyroid gland showing a lower-middle mass with a regular shape and clear margins. (B) Ultrasound of the right lobe of the thyroid gland showing a lower mass with indistinct borders, irregular shape and internal calcified spots.

The patient thyroid ultrasound report indicated that the size and shape of both the right and left lobes of the thyroid gland were within normal range. The echogenicity of the gland was hyperechoic, thickened, and poorly distributed. No significant increase in blood flow signal was observed on color doppler flow imaging (CDFI). Multiple nodules were detected in both lobes. The larger nodule in the left lobe, measuring approximately 4.0 × 2.9 mm, was located in the middle and lower part of the thyroid. It exhibited clear margins, a regular shape, and a cystic nature. In the right lobe, a larger nodule, measuring approximately 6.2 × 6.3 mm, was located in the lower portion. It had an unclear border, an irregular shape, uneven internal echogenicity, no distinct peritoneum, substantial hypoechoi city, and fine punctate calcified echogenicity within the nodule. Punctate blood flow signals were observed at the edge of the nodule on CDFI. Additionally, a neck scan revealed multiple small lymph node echoes bilaterally, exhibiting clear borders and no apparent abnormalities in internal structure. Figure 1A and B display the ultrasound images of the patient right and left thyroid lobes, respectively.

In the preoperative multi-row computed tomography (CT) scan of the chest, a low-density plaque shadow was identified within the right lobe of the thyroid gland. This finding aligns with the presence of right thyroid cancer, as initially detected in the preceding ultrasound examination. As for thyroglossal duct cancer, we did not find it on CDFI and CT examination.

### 2.3. Surgery

**Figure 2.**
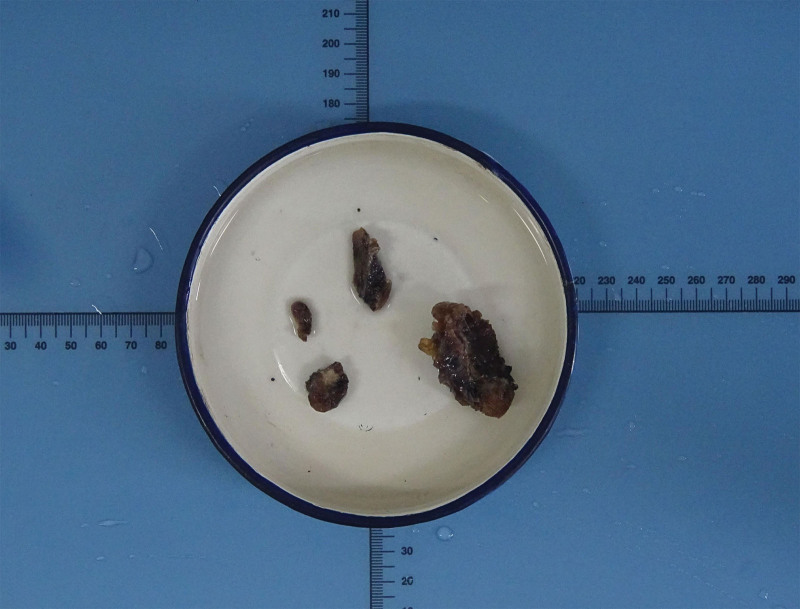
Gross observation of thyroglossal duct cancer after nanocarbon tracing (The part in the red box is the right lobe of the thyroid gland, the part in the blue box is thyroid duct cancer, and the remaining 2 are divided into thyroid tissue and lymph nodes).

Based on preoperative fine-needle aspiration cytology (FNAC), the patient was diagnosed with right-sided thyroid cancer. Additionally, TGDCs were identified during intraoperative exploration. The cyst was meticulously excised through fine perineural dissection. Rapid pathology assessment indicated the presence of thyroglossal duct carcinoma, no cancer or lymph node infiltration was observed at the incisal margin of the tumor, prompting the subsequent performance of a Sistrunk operation and right-sided thyroidectomy. The entire thyroglossal duct carcinoma and the ipsilateral thyroid gland were successfully resected, as depicted in Figure 2, which illustrates the gross anatomy.

### 2.4. Histopathological findings

**Figure 3.**
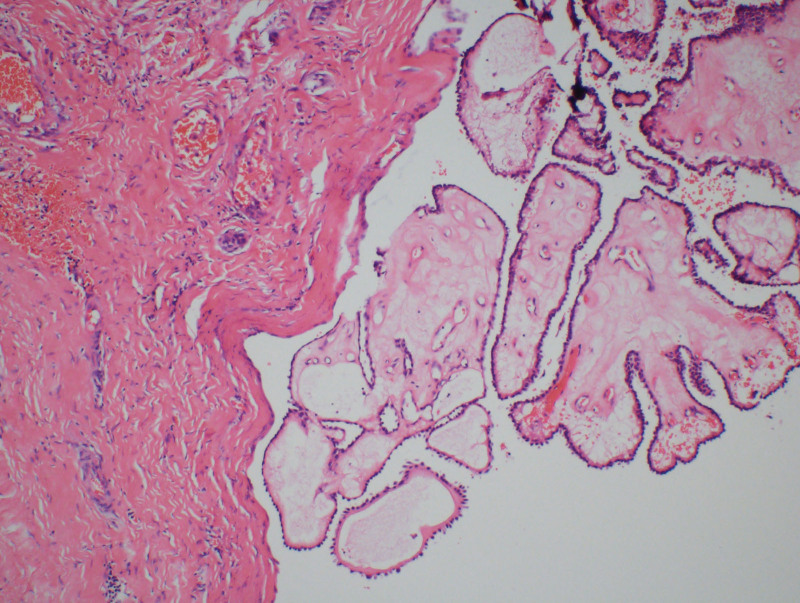
HE (100×) stained section of thyroglossal duct cancer.

During the operation, the TGDC tissue was examined, and ectopic thyroid tissue was found in the tissue, and thyroid papillary carcinoma was found. Thyroglossal duct carcinoma was confirmed by rapid intraoperative histopathological examination. Following a joint diagnosis by 2 experienced pathologists at our hospital, postoperative comprehensive pathology evaluation revealed the following findings: During the surgery, a thyroglossal cyst measuring 2 cm × 1.3 cm × 1.2 cm was submitted for examination. It exhibited localized papillary features. Furthermore, a section of the thyroid gland, displaying swelling and measuring 4 cm × 2.4 cm × 2 cm, revealed the presence of a gray-white nodule. The pathological diagnosis indicated the presence of a micropapillary thyroid carcinoma measuring 0.6 cm in diameter. The carcinoma displayed infiltrative growth, invading the peritoneum of the thyroid gland, and was accompanied by nodular goiter. No metastasis was observed in the local lymph nodes. The final diagnosis was pT1aN0. Figure 3 depicts the results of the HE (100×) staining. For another 2 images of HE stained sections (400×), see Supplementary Figure 1, http://links.lww.com/MD/M906 and Supplementary Figure 2, http://links.lww.com/MD/M907.

### 2.5. Postoperative course

The patient exhibited a satisfactory postoperative recovery and was discharged from the hospital 3 days after the surgery. During the first 3 postoperative days, the amount of drainage from the neck was recorded as 40 mL, 30 mL, and 20 mL, respectively. The healing of the neck incision was deemed satisfactory. After discharge, thyroid function, blood calcium, blood phosphorus and thyroid color ultrasound were recommended to be reexamined 1, 3, and 6 months after surgery, and the dosage of thyroxine was adjusted according to the examination results. At a 3-month follow-up, the patient remains in good health, with no significant increase observed in the size of the left thyroid nodule. Consequently, the patient was satisfied with the course of treatment and continued to be closely monitored.

## 3. Discussion

### 3.1. Epidemiology and etiology

TGDCs are the most prevalent congenital neck malformations, constituting 7% of central neck swellings in adults,^[4]^ and 75% of central neck swellings in children.^[5]^ The overwhelming majority of thyroglossal cysts are benign, with malignant cases accounting for approximately 1%.^[6]^ Ectopic thyroid, characterized by the presence of functional thyroid tissue outside its normal location, typically arises from the abnormal descent of primitive thyroid tissue during embryogenesis.^[7]^ The incidence of this condition is estimated to be around 1:100,000 to 1:300,000.^[8]^ Among ectopic thyroid carcinomas, thyroglossal duct carcinoma stands as the most common clinical type, occurring at a rate of 0.7% to 1.5%.^[9]^ The first reported case of this disease dates back to 1911, documented by Brentano.^[2]^ Papillary carcinomas represent the primary pathological type, followed by mixed papillary/follicular carcinomas, squamous cell carcinomas, follicular carcinomas, and mesenchymal carcinomas.^[10]^ Typically, the disease manifests in individuals in their forties, with a slight predilection for males over females.^[11]^ The likelihood of encountering thyroglossal duct carcinoma alongside carcinoma of the ipsilateral thyroid gland ranges from 25% to 56%.^[12]^

The pathogenesis of thyroglossal duct carcinoma is closely associated with the development of the thyroid gland during embryonic stages. The thyroid gland originates from the proliferation of endodermal epithelial cells in the primitive pharyngeal base around the fourth week of embryonic development. Between 5 and 7 weeks of gestation, the primitive thyroid gland gradually descends through the thyroglossal duct until it reaches the anterolateral aspect of the upper trachea.^[7]^ The thyroglossal duct serves as a slender tube connecting the thyroid gland during its descent. Normally, the thyroglossal duct starts to undergo atresia and degeneration in the embryo by 6 weeks, with complete atresia occurring by 8 weeks. However, in certain embryos, the thyroglossal duct fails to degenerate, leading to the formation of localized cysts situated at the level of the hyoid bone and thyroid cartilage.^[13]^ The most frequent site for thyroglossal cyst development is between the thyroid gland and the hyoid bone, accounting for 61% of cases, followed by the supraglossal (24%), suprasternal (13%), and intralingual (2%) regions.^[14]^ In some instances, the thyroid glands do not descend during the embryonic period and remain within the cysts formed by the original thyroglossal ducts. Subsequently, these aberrant thyroid tissues may develop into ectopic thyroid adenocarcinomas.^[15]^

### 3.2. Clinical presentation

Throglossal duct carcinoma does not typically present with specific manifestations, and clinical symptoms primarily depend on the location and size of the tumor. Alongside the physical examination findings of thyroglossal cysts, patients often seek medical attention due to tracheal compression resulting from rapid tumor growth within a short period.^[16]^ In most cases, patients solely experience neck swelling without any additional symptoms. However, the presence of pain, voice changes, accelerated tumor progression, weight loss, lymphadenopathy, and respiratory symptoms (such as airway compression) often indicate malignant transformation of the cyst. Nevertheless, these symptoms are only observed in a small percentage of patients,^[17]^, which typically correlates with a poor prognosis.

### 3.3. Diagnosis and radiological characteristic

Throglossal duct carcinoma is relatively uncommon in clinical practice, and there is limited literature regarding specific diagnostic criteria for this disease. Pathological examination methods form the primary basis for diagnosis, with the presence of deeply stained nuclei and intracytoplasmic inclusion bodies being key indicators.^[18]^

Currently, the main clinical diagnostic modalities include thyroid ultrasound, neck CT scan, thyroid function tests, and thyroid FNAC. FNAC results are typically considered the gold standard for clinical diagnosis. It is important to note that when performing a puncture on thyroglossal cysts, the presence of intracystic fluid may result in less heterogeneous cells being detected, which can impact the effectiveness of the puncture procedure.^[19]^ Therefore, it is recommended that if ultrasound shows thyroglossal cysts containing a significant amount of cystic fluid, the intracystic fluid should be aspirated prior to the puncture biopsy. Additionally, simultaneous puncture biopsy can be performed on any abnormal nodules found in the ipsilateral thyroid to determine the appropriate surgical approach. Preoperative mutation testing for BRAF V600E is conducted, and a positive result often suggests malignancy of the thyroglossal cyst, frequently associated with lymph node metastasis.^[20]^

Ultrasound diagnosis can provide valuable information about the cyst anatomy, extent, presence of wall nodules, and calcifications. The “claw sign,” which refers to a midline cystic neck mass embedded in the sublingual muscle band, is particularly indicative on ultrasound.^[21]^ Patients presenting with a thyroglossal cyst that exhibits solid nodules, calcifications, thickening of the cyst wall, irregular shape of the cyst wall, and abundant blood supply should be alerted to the possibility of thyroglossal duct carcinoma.^[16,22]^

### 3.4. Treatment

Given the rarity of thyroglossal duct carcinoma, there is currently no established standard treatment guideline for this condition. Treatment approaches typically involve a combination of surgery and radiotherapy.

After the initial diagnosis of a thyroglossal cyst, FNAC is feasible. If the FNAC shows a simple cyst, the Sistrunk procedure is performed. Sistrunk surgery was performed if rapid pathology showed thyroglossal duct carcinoma and the following conditions were met: intact tumor envelope, normal thyroid morphology, age < 45 years, tumor < 4 cm, and negative for BRAF gene. Postoperative pathology required no further surgery if it showed complete resection of the cancer; if it showed residual cancer or unclear borders, additional ipsilateral thyroidectomy was required.

**Figure 4.**
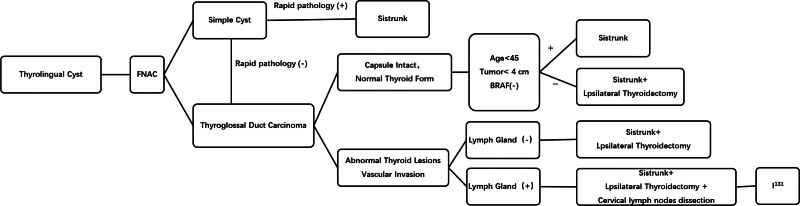
Thyroglossal cyst diagnosis and treatment flowchart.

Sistrunk surgery with additional ipsilateral thyroidectomy was performed if the patient was ≥ 45 years old, had a tumor ≥ 4 cm, or was BRAF gene positive. If pathology showed abnormal thyroid lesions or vascular invasion, further lymph node examination was required. If the lymph nodes are negative, the Sistrunk procedure with additional ipsilateral thyroidectomy is performed; if the lymph nodes are positive, the Sistrunk procedure, ipsilateral thyroidectomy, and cervical lymph node dissection are performed. After cervical lymph node dissection, the need for radioactive iodine therapy to further remove residual cancer cells will be determined based on postoperative pathology and disease assessment, as depicted in Figure 4.

#### 3.4.1. Operative treatment

The main surgical approaches for thyroglossal duct carcinoma depend on its origin and include the Sistrunk procedure, which involves the excision of the thyroglossal cyst, and the combined Sistrunk procedure with ipsilateral thyroidectomy. The former is primarily employed for the treatment of malignant transformation of the ectopic thyroid gland within the thyroglossal cyst and is the main surgical procedure performed in clinical practice with a low postoperative recurrence rate of only 3%.^[23]^ The key steps of this procedure include excising the cyst and cancerous tissue, removing the hyoid bone, and eliminating the core tissue around the lingual bundle of the thyroid near the blind foramen from the lingual muscle.^[24]^ The latter approach is typically used in cases where there is metastasis of ipsilateral thyroid cancer to the ectopic thyroid gland within the ipsilateral thyroglossal hyoid bone or in the presence of multiple thyroid cancers.^[25,26]^ In the current patient case, no obvious clinical symptoms or lymph node metastasis were observed during physical examination, and the patient was in an early clinical stage. Therefore, only the combined Sistrunk procedure with ipsilateral thyroidectomy was performed. It has been reported that approximately 7.7% to 12.9% of patients with main symptoms of neck compression, hoarseness, and dyspnea exhibit cervical lymph node metastases. If existing cervical lymph node metastases are detected or abnormal lymph node structures are observed on cervical imaging, cervical lymph node dissection should be performed.^[3]^ Currently, it is generally recommended to perform grade-separated surgery, with the surgical approach determined based on the patient high-risk factors. High-risk factors include age over 45 years, the presence of a cold nodule on thyroid scan, positive tumor margins, tumor size larger than 4 cm, lymph node metastases, and a history of cervical radiotherapy. In such cases, routine performance of the combined Sistrunk procedure with ipsilateral thyroidectomy and lymph node dissection is recommended, whereas patients without high-risk factors typically undergo the Sistrunk procedure alone.^[13,27,28]^ Prophylactic cervical lymph node dissection is generally not recommended, but prophylactic central zone VI lymph node dissection may be considered.^[29]^ If preoperative laboratory testing reveals the presence of BRAF V600E mutation, it is usually associated with a poor prognosis, and resection should be extended as much as possible, avoiding the use of the Sistrunk procedure alone.^[30,31]^

#### 3.4.2. Radiotherapy

The need for I^131^ treatment after thyroglossal duct cancer is still controversial. In the available retrospective studies, some researchers believe that I^131^ treatment actually worsens the prognosis of patients.^[11]^ Currently, radioactive iodine therapy is only recommended for thyroglossal duct cancer in combination with ipsilateral thyroid cancer and lymph node metastases.^[23]^ The most common genetic mutation in thyroid cancer is the BRAF mutation, which accounts for about 45% of papillary thyroid cancers,^[32]^ and the most common BRAF family variant in thyroid cancer is BRAF V600E in the BRAF1 gene. Some studies have also shown that patients with BRAF mutations should be routinely treated with I^131^ after surgery to reduce the risk of recurrence.^[33]^

### 3.5. Prognosis

Thyroglossal duct carcinoma generally has a favorable prognosis, except in cases where the patient tests positive for the BRAF V600E mutation.^[34]^ The recurrence rate after complete resection of the lesion is <5%.^[35]^ Among all types of thyroglossal duct carcinoma, squamous cell carcinoma carries the worst prognosis.^[36]^ The presence of significant clinical symptoms at the time of diagnosis may indicate a good prognosis and a lower likelihood of recurrence.^[37]^

## 4. Conclusion

The patient described in this article had a tumor located within a cyst formed by the undeveloped thyroglossal duct. There was evidence of local peritumoral infiltration but no metastasis to the surrounding cervical lymph nodes or distant sites. The tumor, along with the TGDC and ipsilateral thyroid lobe, was completely removed during surgery. Following the procedure, the patient was discharged with oral thyroid medication and scheduled for regular follow-up.

Although the patient had a BRAF V600E gene mutation, there was no lymph node infiltration observed locally. Therefore, if thyroid cancer is detected during follow-up, prophylactic I^131^ treatment can be administered.

In this study, we found the presence of BRAF V600E mutation, and we believe that the detection of BRAF V600E mutation can be used as an auxiliary diagnostic tool for thyroglossal carcinoma, which can improve the diagnostic accuracy and early detection rate. Although the study of BRAF V600E mutation in thyroglossal carcinoma is still in the early stages, its potential clinical application is promising. Future studies should further investigate the incidence, molecular mechanism and relationship between BRAF V600E mutation and other molecular markers in thyroglossal duct carcinoma.

In conclusion, thyroglossal duct carcinoma is a rare ectopic thyroid tumor that is often challenging to diagnose preoperatively. Early clinical stages typically lack specific indicators, and most patients present with nonspecific symptoms such as tumor compression. The overall prognosis for this disease is favorable. This paper outlines a diagnostic and therapeutic flowchart based on existing literature and clinical experience, although further support from relevant clinical trials may be necessary to address its limitations.

## Author contributions

**Conceptualization:** Guang Chen.

**Data curation:** Fan Bu.

**Formal analysis:** Kai Yu, Fang Wan.

**Investigation:** Bingfei Dong.

**Methodology:** Ji Lu.

**Project administration:** Shuai Xue.

**Resources:** Dandan Yu.

**Supervision:** Jixue Wang.

**Validation:** Li Rong.

**Visualization:** Wenjun Wang.

## Supplementary Material




