# Supplementary figures and images for: Research progress of ectopic thyroid cancer in thyroglossal duct cyst: A case report and literature review
Source: Medicine (Baltimore). 2024 Jun 28;103(26):e38540. doi: 10.1097/MD.0000000000038540 (PMC11466096; doi:10.1097/MD.0000000000038540)

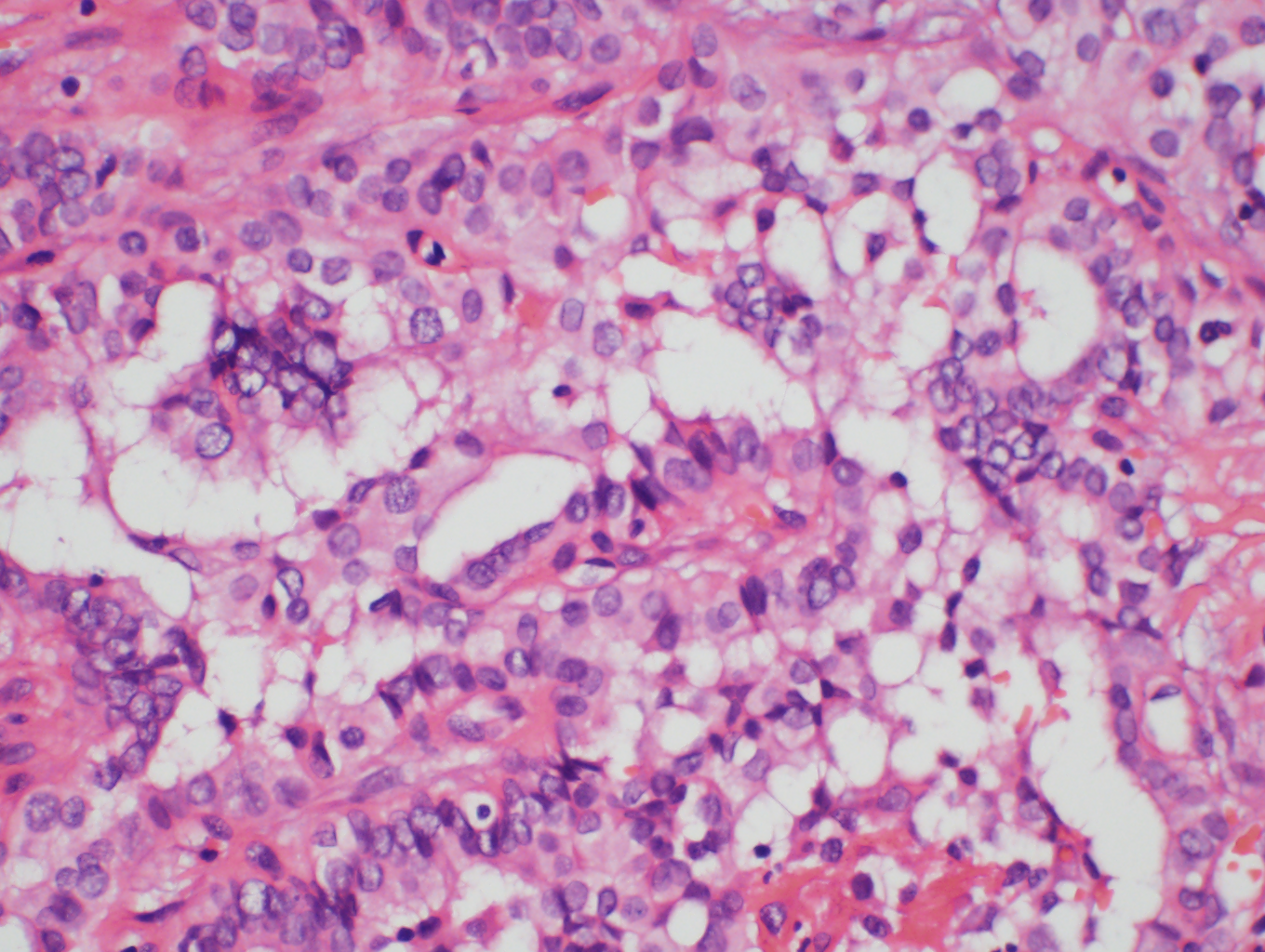

Supplement: Supplementary file 1 [file medi-103-e38540-s001.tif]

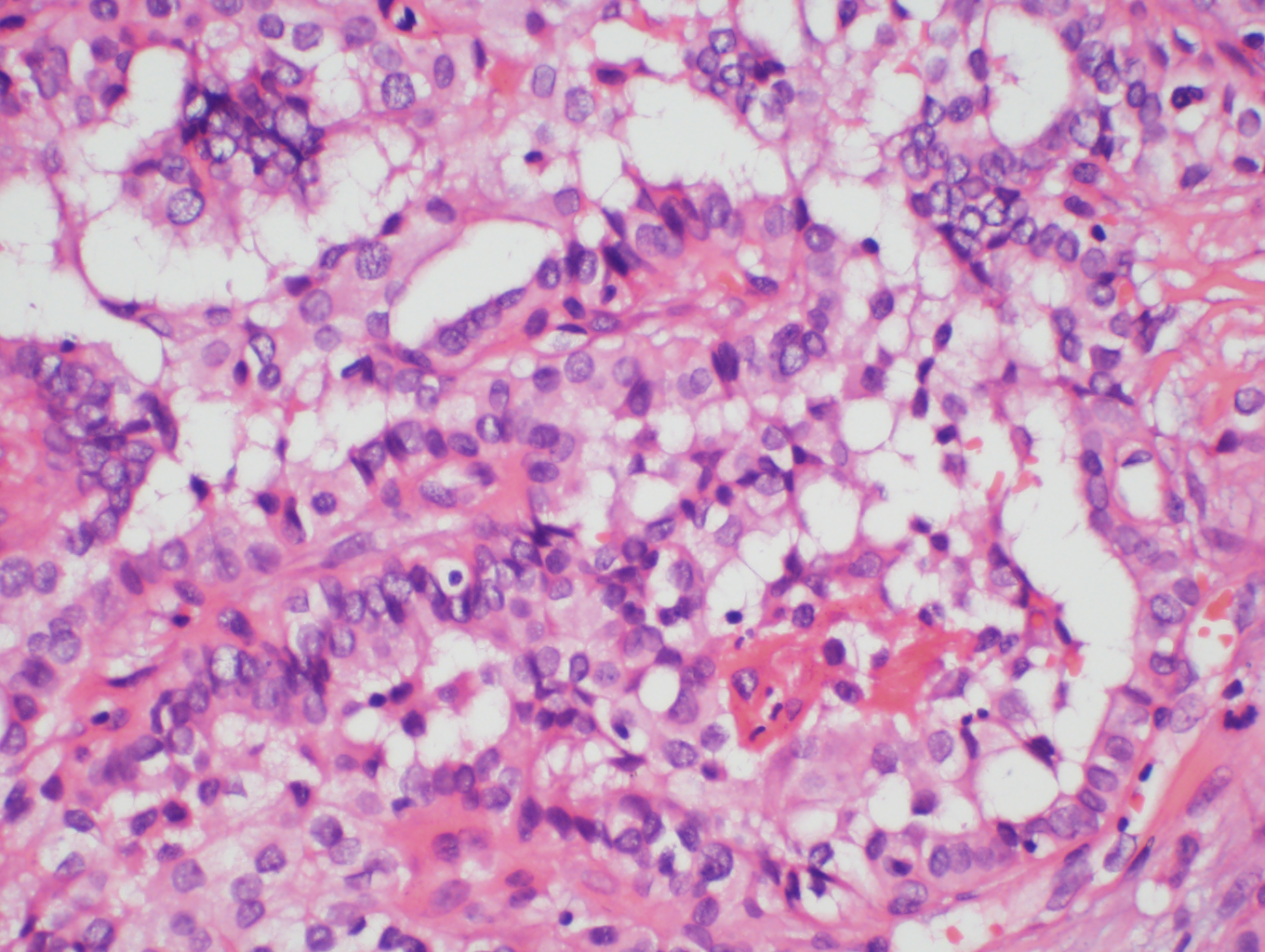

Supplement: Supplementary file 2 [file medi-103-e38540-s002.tif]
